# How to Face COVID-19 Outbreak: Reconfiguration of a Private Radiological Clinic

**DOI:** 10.34172/ijhpm.2020.165

**Published:** 2020-08-26

**Authors:** Michaela Cellina, Filippo Pesapane, Laura Bracchi, Gianfranco Bracchi, Anna Maria Ierardi, Carlo Martinenghi, Gianpaolo Carrafiello

**Affiliations:** ^1^Department of Radiology, ASST Fatebenefratelli Sacco, Milan, Italy.; ^2^University of Milan, Milan, Italy.; ^3^Cerba Healthcare Italia, Milan, Italy.; ^4^Radiology Department, Fondazione IRCCS Cà Granda Ospedale Maggiore Policlinico, Milan, Italy

## Dear Editor,

Radiology Units have been involved in this emergency, to provide lung imaging assessment in coronavirus disease 2019 (COVID-19) patients.^1^ At the same time, diagnostic imaging facilities must maintain standard radiologic support for other patients. Although cancer patients showed a higher risk of COVID-19 infection and a poorer prognosis,^2^ regular follow-up should be executed guaranteeing patients and staff safety through dedicated pathways.^3,4^ Therefore, a reconfiguration of Radiology Units with the application of infection control procedures and protocols to manage subjects with suspected COVID-19 infection is essential.

 We report the reorganization of a private radiological clinic in Lombardy, the epicenter of the outbreak in Italy.

 The reconfiguration was divided into several levels.

 Checkpoints for temperature measurements of all outpatients and staff have been set at the entrances, where some posters explain the procedures in place in the center, namely the mandatory maintenance of the safe distance and the restriction of access of patient’s companion/s. All patients are informed that, in the presence of suspected symptoms, only chest imaging will be performed.

At the front desk, where the staff is located behind a glass barrier protection (Figure a), the patient receives a surgical mask, and fills out a form on the epidemiological history and cough and flu-like symptoms. Patients with no exposure history or symptoms seat in the waiting room, where the chairs are positioned at a safe distance.^3^

Patients reporting symptoms or with a temperature >37.2°C are classified as “high risk for COVID-19 infection.”^3^ The COVID-19 dedicated pathway is distinct from that of the other patients, indicated with directional signs, and isolated using physical barricades.^4^ High-risk patients do not enter the waiting room but are referred directly to the X-ray or computed tomography (CT) Units, according with the general practitioners’ requests. Radiologic technologist (RTs) are supplied with appropriate personal protective equipment (PPE), and trained for its use (Figure b, c). In the CT suite, one RT with PPE sets up the patient position on the table, whereas a second one, with a surgical mask and disposable gloves, operates the machine console.^6,7^

**Figure:**
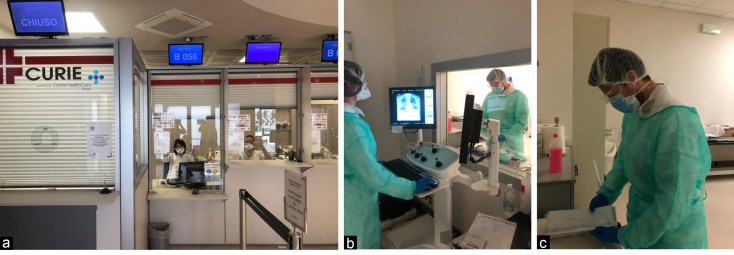
(a) Staff at the front desk is now behind a glass barrier protection, to avoid direct contact with the patients. (b, c) The RT wear a complete PPE: hair cap, goggles for eye protection, disposable long sleeve fluid-resistant gown, two pairs of disposable gloves, and a double face mask, a FFP2 below and a surgical mask above, over goggles (c).^6^ Abbreviation: PPE, personal protective equipment.

 After the imaging acquisition, the patient waits in a dedicated waiting room while the radiologist urgently reports the examination. If no imaging features suspected for COVID-19 infection are observed, the patient can leave. In the case of abnormal findings, the radiologist calls the patient’s General Practitioner to communicate the examination results; the patient leaves the clinic via a dedicated pathway and exit and is referred to a reference laboratory to perform nasopharyngeal swab.

 In the case of incidental detection of findings suggestive for COVID-19 pneumonia in asymptomatic cases, the patients and their General Practitioners are promptly contacted.

After each exam of suspected COVID-19 patients, the CT and X-Ray rooms are closed for the next thirty minutes to exchange the air.^8,9^ In this time, the RT, protected with PPE, cleans contact surfaces and the radiological consoles with a cloth soaked with alcohol-based disinfectants (75% ethanol), then carefully removes PPE to avoid self-contamination, starting from the removal of the first pair of gloves, followed by the gown under the supervision of another colleague.^10^ Used PPE is disposed of in special waste.

The ground in X-ray, CT and waiting rooms is wiped with 1000 mg/L chlorine-containing disinfectant, once every 4 hours, by the cleaning company staff.^8,9^

The measures to face an infectious disease outbreak are determined by the estimated risk of cross-infection to the staff and other patients.^10^ When the risk is high, as in the current case of COVID-19, due to the transmission modality and high prevalence in the population, strict control protocols need to be applied, including the creation of dedicated pathways, to limit the risk of patient-to-patient transmission, the supply of adequate PPE and anti-infection training of the staff, to protect the staff members, and disinfection procedures, to avoid patient-to-patient transmission.^8-10^ All healthcare workers undergo serological tests for COVID-19 antibodies research once a month and no cases of infection among staff members has been diagnosed so far.

 Our experience in management, with reconfiguration of the clinic and application of an innovative protocol can be useful for other radiology departments and private radiological clinics dealing with this pandemic. Particularly, the staff training, correct use of PPE, and disinfection procedures are the key points to avoid the spread of COVID-19 infection.

## Ethical issues

 Not applicable.

## Competing interests

 Authors declare that they have no competing interests.

## Authors’ contributions

 Conception and design of the study: GC, LB. Acquisition of data: GB. Analysis and interpretation of the data: AMI. Drafting the article: MC, FP. Revising draft critically for important intellectual content: CM, MC. Administrative, technical, material support: GC. Supervision: GC.

## Authors’ affiliations

^1^Department of Radiology, ASST Fatebenefratelli Sacco, Milan, Italy. ^2^University of Milan, Milan, Italy. ^3^Cerba Healthcare Italia, Milan, Italy. ^4^Radiology Department, Fondazione IRCCS Cà Granda Ospedale Maggiore Policlinico, Milan, Italy.
